# Two-sample mendelian randomization analysis investigates ambient fine particulate matter's impact on cardiovascular disease development

**DOI:** 10.1038/s41598-023-46816-3

**Published:** 2023-11-17

**Authors:** Xiao Liang, Lianjing Liang, Yuchao Fan

**Affiliations:** 1 Department of Anesthesiology, West China Hospital, Sichuan University, Chengdu, Sichuan Province China; 2 Department of Emergency Medicine, West China Hospital, Sichuan University, Chengdu, Sichuan Province China; 3 Department of Anesthesiology, Sichuan Clinical Research Center for Cancer, Sichuan Cancer Hospital and Institute, Sichuan Cancer Center, Affiliated Cancer Hospital of the University of Electronic Science and Technology of China, No. 55, Section 4, Renmin South Road, Chengdu, 610041 Sichuan Province China

**Keywords:** Environmental impact, Genetics, Environmental sciences, Cardiology, Diseases, Risk factors, Disease prevention, Public health

## Abstract

PM2.5, a key component of air pollution, significantly threatens public health. Cardiovascular disease is increasingly associated with air pollution, necessitating more research. This study used a meticulous two-sample Mendelian randomization (MR) approach to investigate the potential causal link between elevated PM2.5 levels and 25 types of cardiovascular diseases. Data sourced from the UK Biobank, focusing on individuals of European ancestry, underwent primary analysis using Inverse Variance Weighting. Additional methods such as MR-Egger, weighted median, Simple mode, and Weighted mode provided support. Sensitivity analyses assessed instrument variable heterogeneity, pleiotropy, and potential weak instrument variables. The study revealed a causal link between PM2.5 exposure and higher diagnoses of Atherosclerotic heart disease (primary or secondary, OR [95% CI] 1.0307 [1.0103–1.0516], p-value = 0.003 and OR [95% CI] 1.0179 [1.0028–1.0333], p-value = 0.0202) and Angina pectoris (primary or secondary, OR [95% CI] 1.0303 [1.0160–1.0449], p-value = 3.04e−05 and OR [95% CI] 1.0339 [1.0081–1.0603], p-value = 0.0096). Additionally, PM2.5 exposure increased the likelihood of diagnoses like Other forms of chronic ischaemic heart disease (secondary, OR [95% CI] 1.0193 [1.0042–1.0346], p-value = 0.0121), Essential hypertension (secondary, OR [95% CI] 1.0567 [1.0142–1.1010], p-value = 0.0085), Palpitations (OR [95% CI] 1.0163 [1.0071–1.0257], p-value = 5e−04), and Stroke (OR [95% CI] 1.0208 [1.0020–1.0401], p-value = 0.0301). Rigorous sensitivity analyses confirmed these significant findings' robustness and validity. Our study revealed the causal effect between higher PM2.5 concentrations and increased cardiovascular disease risks. This evidence is vital for policymakers and healthcare providers, urging targeted interventions to reduce PM2.5 levels.

## Introduction

Air pollution stands as a formidable global public health challenge due to its adverse effects on personal well-being and socio-economic factors^1^. Evidence reveals that approximately 90% of the world's population is exposed to air pollution, leading to an estimated nine million premature deaths in 2015 and making it a predominant cause of global premature mortality^2,3^. Among the array of pollutants, fine particulate matter, specifically Particulate Matter (PM) 2.5, constitutes a substantial portion, emanating chiefly from sources such as coal combustion, vehicular discharges, and industrial activities^4^. Crucially, it is noteworthy that even if PM2.5 concentrations fall below the thresholds set by the World Health Organization (WHO) and the US Environmental Protection Agency, potential health risks persist^5,6^. Alarmingly, PM2.5 concentrations surpass WHO standards in 90% of global regions^7^. The impact of PM2.5 is staggering, resulting in a significant toll of 80 to 420 million attributable deaths and considerable economic losses. Notably, the elderly population bears the brunt, with PM2.5-induced deaths accounting for 59% of the total population cost^8^. PM2.5 pollution exacts a heavy toll on human capital, productivity, and social welfare, curtailing life expectancy and underscoring the urgent need for comprehensive interventions to mitigate this pervasive environmental hazard.

Cardiovascular disease stands as the foremost global cause of mortality, marked by a high incidence rate and substantial healthcare expenditures^9^. The etiology of cardiovascular disease is multifaceted, encompassing genetic predisposition, age, lifestyle, infections, and environmental influences, the latter of which are progressively gaining prominence. An accumulating body of evidence underscores the pivotal role of air pollution not only in exacerbating the trajectory of cardiovascular disease but also in fostering its inception^10^. Indeed, air pollution emerges as the paramount environmental determinant of cardiovascular disease-related fatalities, contributing to more than half of all deaths linked to air pollution^11^. Among the diverse array of airborne contaminants, PM exhibits the most robust and clinically significant association with cardiovascular well-being, with specific emphasis on PM2.5. The diminutive size of PM2.5 facilitates its evasion of the body's respiratory defense mechanisms, enabling its intrusion into the bloodstream and inciting localized and systemic inflammation, thereby exerting a profound influence on cardiovascular health^12^. Both transient and sustained exposure to PM2.5 have been intimately associated with heightened incidence rates and mortality rates for cardiovascular disease. Prolonged PM2.5 exposure is correlated with an elevated risk of diverse cardiovascular diseases, including coronary heart disease, myocardial infarction (MI), heart failure, arrhythmia, and other related conditions^13^.

Although several observational studies have indicated an association between PM2.5 and cardiovascular disease^2,14,15^, additional evidence is imperative to establish a stronger causal relationship. This necessity arises from potential confounding, misclassification, and the inherent challenges of reverse causality prevalent in observational study design^16,17^. To surmount these issues, Mendelian randomization (MR) employing Genome-Wide Association Study (GWAS) summary statistics has emerged as a powerful tool for inferring causality in complex diseases^17^. The rationale behind MR analysis rests on the random allocation of genetic variation inherited from parents at conception, akin to the random assignment of participants to different experimental groups in a randomized controlled trial^18^. In the present study, we adopted a two-sample MR approach to rigorously investigate the causal impact of elevated PM2.5 levels on the incidence of cardiovascular diseases.

## Methods

### Study design

This study utilizes a two-sample MR approach, which employs summary statistics from GWAS, to investigate the potential causal relationship between levels of PM2.5 and the risk of multiple cardiovascular diseases. To satisfy the assumptions of instrumental variables (IVs) analysis in MR, single nucleotide polymorphisms (SNPs) were employed to estimate the causal effect of exposure on outcome. SNPs are randomly allocated during meiosis, thereby minimizing classical forms of bias observed in observational studies, such as confounding, reverse causality, and measurement error. Therefore, if certain assumptions are met, they can be utilized to investigate causal effects on outcomes. In the MR analyses, PM2.5 levels were considered as the exposure of interest while various cardiovascular diseases were examined as outcomes. The study did not require ethical approval since publicly available data was used. The fundamental prerequisites for genetic variation to meet the IV assumptions in this study are outlined as follows, as indicated in Figure S1: (1) The genetic variant must demonstrate an association with the exposure; (2) the genetic variant must not display an association with any confounding factors that could affect the relationship between the exposure and outcome; and (3) the genetic variant should not directly influence the outcome, except through its association with the exposure.

### Data source

The dataset utilized in this study was obtained from the IEU open GWAS project (https://gwas.mrcieu.ac.uk/), which encompasses data from the UK Biobank study. To control for confounding factors, this study employed genetic associations derived from independent GWAS datasets with the same ancestral population.

The dataset containing information on PM2.5 was acquired from the UK Biobank's Metadata of Environmental Exposures. To estimate PM2.5 levels for the year 2010 at each address, a Land Use Regression (LUR) model developed as part of the European Study of Cohorts for Air Pollution Effects was utilized. The ESCAPE project received funding under the EU 7th Framework Programme. The LUR model is based on monitoring conducted between January 26, 2010, and January 18, 2011, and the resulting air pollution estimates are representative of the year 2010. The dataset consists of 423,796 data items, corresponding to 423,796 participants. The measurement unit used is µg/m3, with a mean value of 9.9919 and a standard deviation of 1.057. The dataset explores genetic associations with increasing levels of PM2.5.

The genetic associations with cardiovascular diseases considered in this study were also obtained from the UK Biobank, which encompasses a summary of main and secondary diagnoses of 25 types of cardiovascular diseases under ICD10 codes of hospital inpatients. The incorporated data types of cardiovascular diseases are binary. A detailed summary of the datasets utilized in this study can be found in Table S1.

### Selection of instrumental variables

The selection process for IVs involved stringent criteria to ensure their validity and reliability. IVs were chosen based on their significant genome-wide association with the exposure (P < 5e−8), a minor allele frequency above 0.01 in the outcome, and low linkage disequilibrium (LD) r2 within a 10,000 kb distance. SNPs associated with confounding factors or outcomes according to the Phenoscanner database were excluded from the study. The proportion of variance explained by individual SNPs was estimated using the formula R2 = 2 × β2 × EAF × (1 − EAF)/(2 × β2 × EAF × (1 − EAF) + 2 × SE2 × N × EAF × (1 − EAF)). R2 in MR analysis represents the "proportion of variance explained." It quantifies the strength of the relationship between genetic variation (used as IVs) and a specific outcome variable, in this case, cardiovascular disease. β2 denotes the regression coefficient, signifying the strength of the association between the IVs and the outcome variable (cardiovascular diseases). EAF, or effect allele frequency, reflects the frequency of genetic variation. SE2 represents the variance of the outcome variable, indicating the extent of variability in the outcome variable (in this context, cardiovascular diseases). It serves as a statistical measure of the outcome variable's distribution. N denotes the number of individuals included in the study.

To assess the strength of the IVs, we computed the F-statistic using the formula F = (N − k − 1)/k × R2/(1 − R2), where N represents the number of samples exposed to the GWAS, k is the number of IVs, and R2 indicates the proportion of exposure explained by the IVs. The F-statistic values were utilized to evaluate the IVs, and instruments with low values (less than 10) were considered weak, potentially introducing bias into the results. Consequently, the study's findings were interpreted cautiously, acknowledging the limitations associated with weak IVs.

### MR analysis

This study employed five distinct methodologies of MR to investigate the causal relationship between exposure and outcome. The primary approach used was the Inverse Variance Weighting (IVW) method, which is renowned for providing precise estimates of causality when all variants function as valid IVs^19^. In addition to IVW, other methods including MR-Egger, weighted median, Simple mode, and Weighted mode were employed. MR-Egger regression was utilized to address horizontal pleiotropy, albeit with slightly reduced precision. Support for MR-Egger was determined if the effect estimate aligned with MR-IVW and the MR-Egger intercept was statistically nonsignificant (P > 0.05). Furthermore, the weighted median method was used as a complementary approach, providing reliable causal estimates as long as at least 50% of the weight in the analysis originated from valid IVs^20^.

To evaluate heterogeneity, the MR-Egger method was employed, utilizing Cochran's Q statistic^21,22^. Heterogeneity was considered absent if the p-value exceeded 0.05. Furthermore, a "leave-one-out" analysis was conducted to assess the impact of individual SNPs on the causal relationship between exposure and outcome. In cases where heterogeneity was detected, a random-effects IVW method was utilized to estimate the causal association, while a fixed-effects model was employed when heterogeneity was not present. To identify potential pleiotropy in the IVs, the MR-Egger intercept and MR-PRESSO tests were utilized^23^. An absence of pleiotropy was indicated by a p-value greater than 0.05. The MR-PRESSO method was used to identify SNP outliers and ensure consistent results with the IVW method after removing these outliers^23^. Associations were considered statistically significant if the P-values in the IVW and MR-PRESSO methods were below 0.05, and the results from MR-Egger, weighted median, Simple mode, and Weighted mode methods aligned with the IVW findings. Effect estimates were reported as odds ratios (ORs) with corresponding 95% confidence intervals (CIs). All MR analyses were performed using R software (version 4.2.2) and the R Packages "TwoSampleMR" and "MRPRESSO." Forest plots depicting the causal effects of PM2.5 exposure on the risk of 25 cardiovascular diseases were generated using the R Package "ggplot2 [3.3.6]."

## Results

Eight SNPs were extracted to investigate the potential causal relationship between PM2.5) increasing and the risk of multiple cardiovascular diseases. These eight SNPs were not associated with cardiovascular diseases or related confounders in Phenoscanner and were therefore treated as IVs.

**Figure 1:**
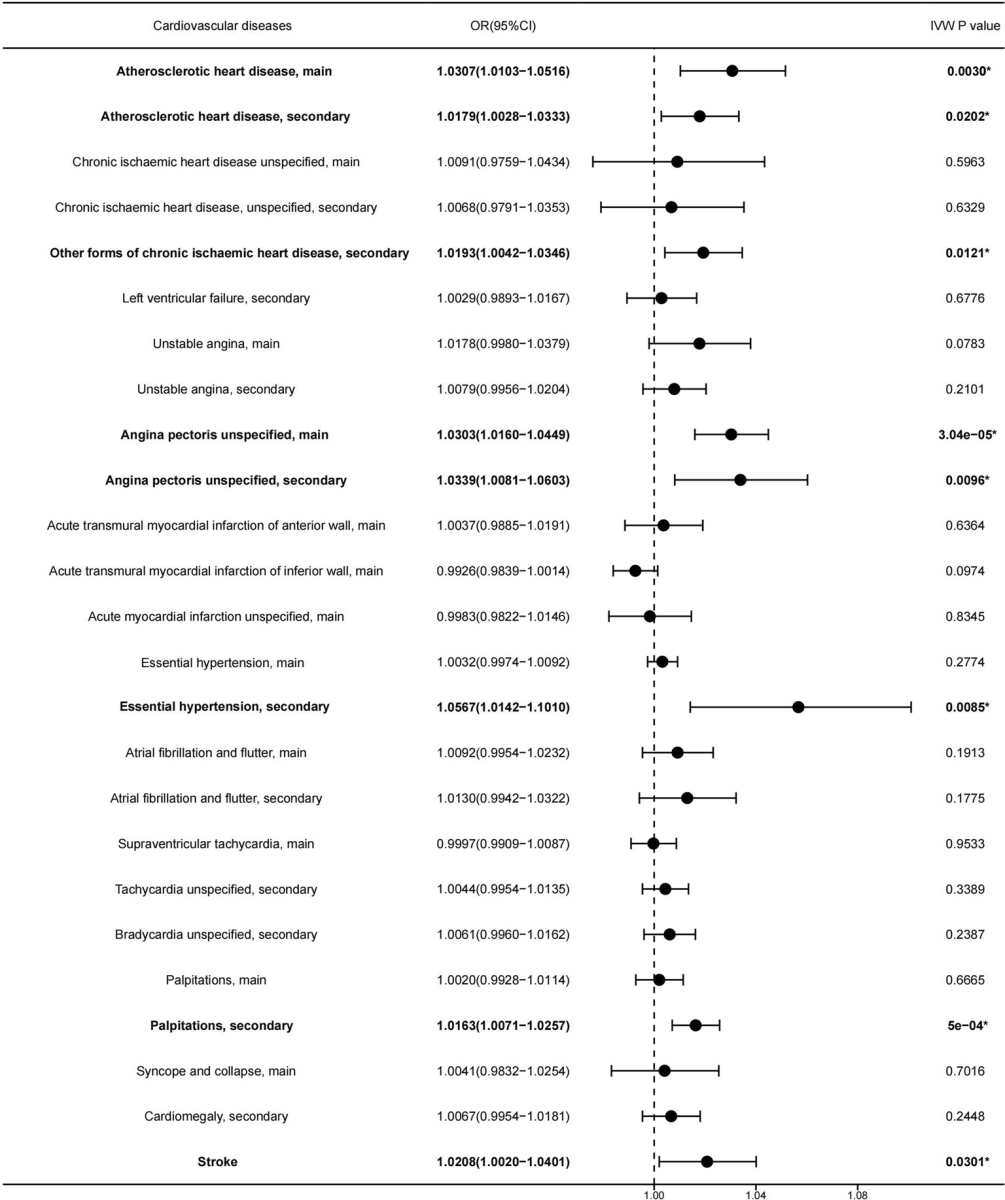
Forest plot of the potential causal association between PM2.5 increasing and risk of 25 types of cardiovascular diseases. PM2.5 increasing was causally associated with a higher risk of cardiovascular diseases, as indicated by its highlighted representation in Bolded font and *.

**Figure 2:**
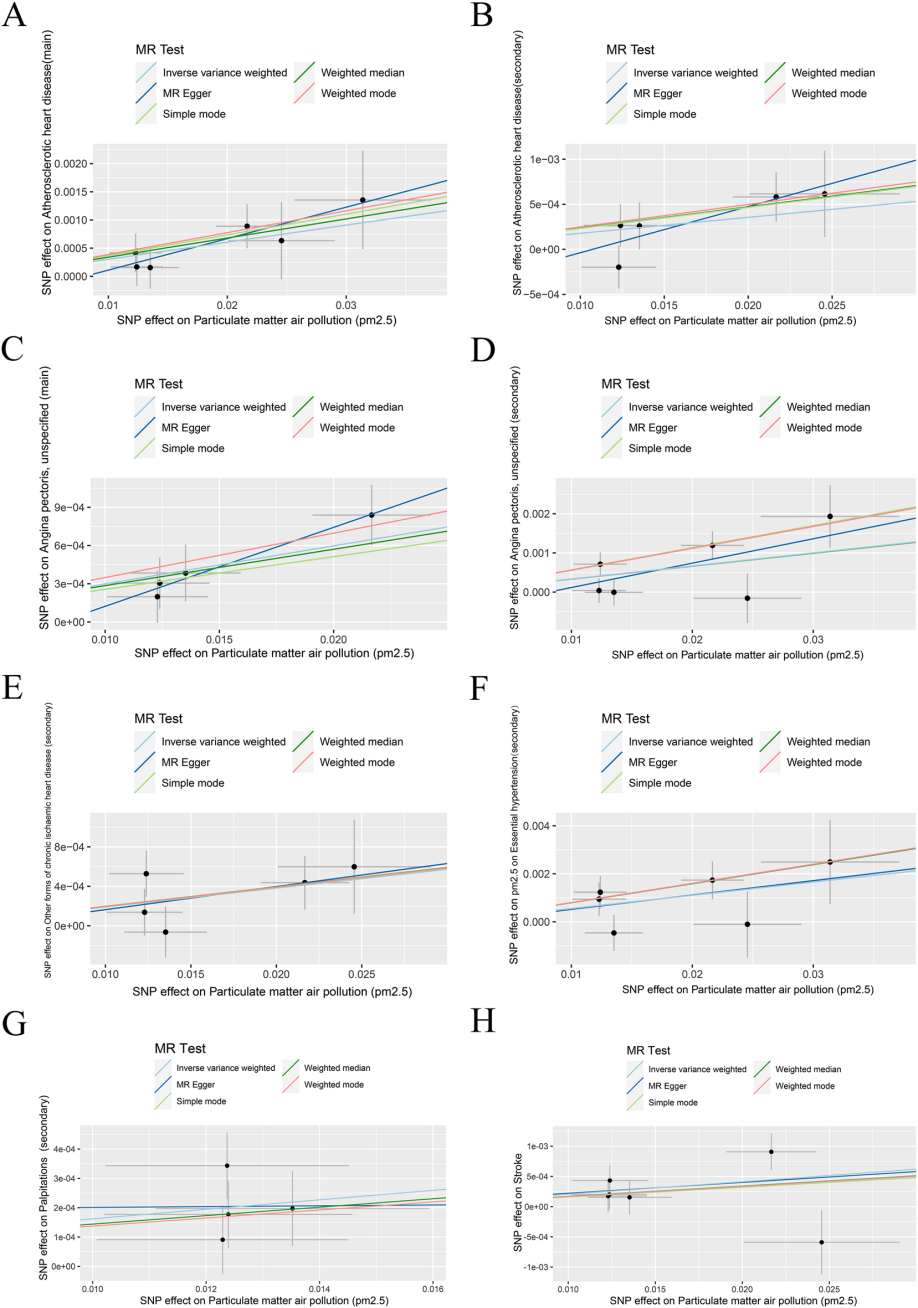
Scatter plot displaying individual Mendelian randomization estimates of the effect of SNPs on PM2.5 increasing and risk of 25 types of cardiovascular diseases. (**A**) Atherosclerotic heart disease, main, (**B**) Atherosclerotic heart disease, secondary, (**C**) Angina pectoris unspecified, main**, **(**D**) Angina pectoris unspecified, secondary**, **(**E**) Other forms of chronic ischaemic heart disease, secondary**, **(**F**) Essential hypertension, secondary, (**G**) Palpitations, secondary**,** and (**H**) Stroke. MR, Mendelian randomization; SNP, single-nucleotide polymorphism.

**Figure 3:**
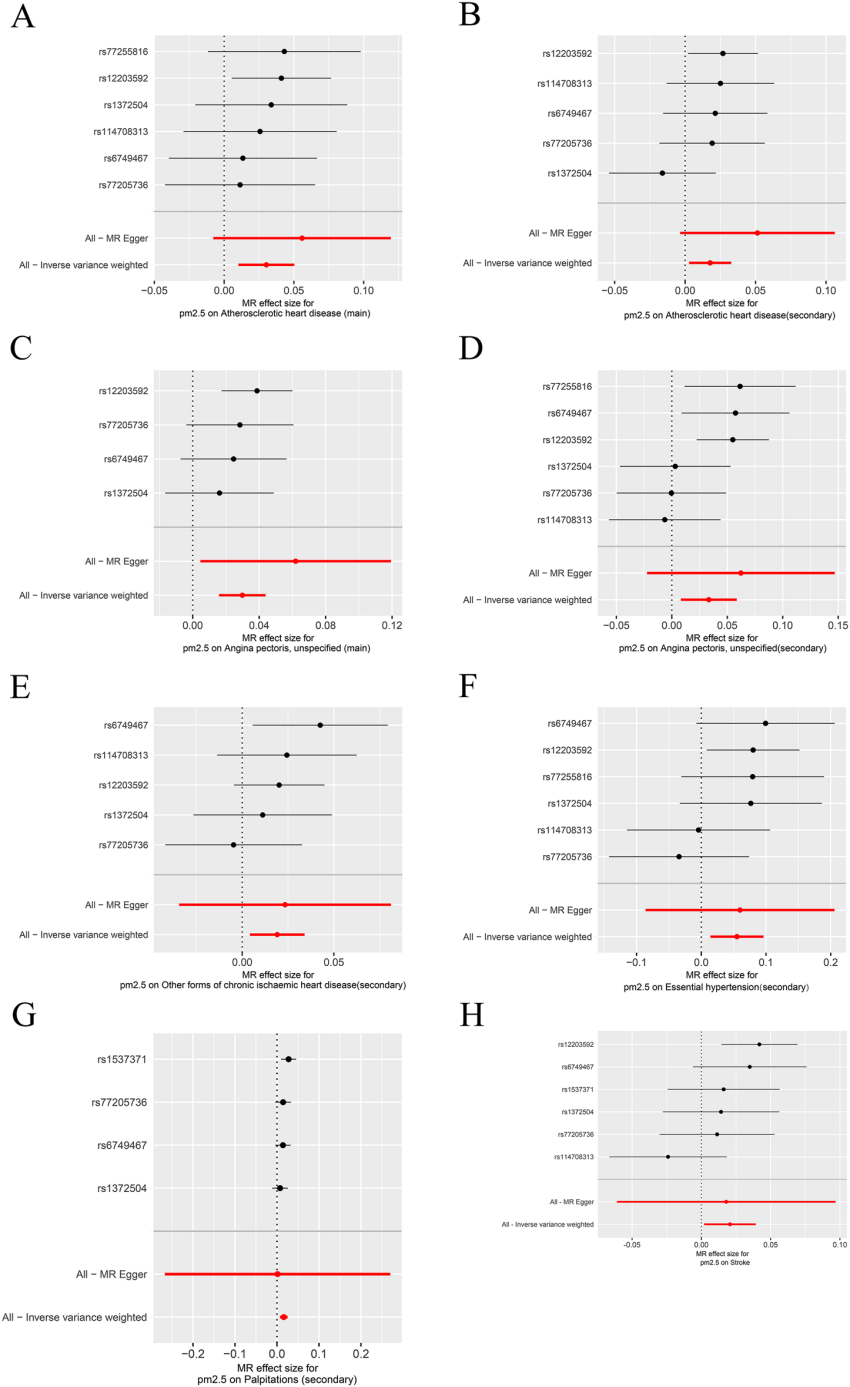
Forest plot illustrating the Mendelian randomization effect size for PM2.5 increasing on risk of 25 types of cardiovascular diseases. (**A**) Atherosclerotic heart disease, main, (**B**) Atherosclerotic heart disease, secondary, (**C**) Angina pectoris unspecified, main**, **(**D**) Angina pectoris unspecified, secondary**, **(**E**) Other forms of chronic ischaemic heart disease, secondary**, **(**F**) Essential hypertension, secondary, (**G**) Palpitations, secondary**,** and (**H**) Stroke. MR, Mendelian randomization; SNP, single-nucleotide polymorphism.

We employed the IVW method as the primary approach to assessing the causal effects. The results found that every increasing 1.057µg/m^3^ of PM2.5 level was causally associated with increased risk of being diagnosed with Atherosclerotic heart disease (ASHD), main(OR [95% CI] 1.0307 [1.0103–1.0516], p-value = 0.003) (Fig. 1, 2A, 3A) , ASHD, secondary (OR [95% CI] 1.0179 [1.0028–1.0333], p-value = 0.0202) (Fig. 1, 2B, 3B), Angina pectoris unspecified, main(OR [95% CI] 1.0303 [1.0016–1.0449], p-value = 3.04e−5) (Fig. 1, 2C, 3C), Angina pectoris unspecified, secondary(OR [95% CI] 1.0339 [1.0081–1.0603], p-value = 0.0096) (Fig. 1, 2D, 3D), Other forms of chronic ischaemic heart disease, secondary(OR [95% CI] 1.0193 [1.0042–1.0346], p-value = 0.0121) (Fig. 1, 2E, 3E), Essential hypertension, secondary (OR [95% CI] 1.0567 [1.0142–1.101], p-value = 0.0085) (Fig. 1, 2F, 3F), Palpitations, secondary(OR [95% CI] 1.0163 [1.0071–1.0257], p-value = 5e−4) (Fig. 1, 2G, 3G), and Stroke (OR [95% CI] 1.0208 [1.0020–1.0401], p-value = 0.0301) (Fig. 1, 2H, 3H).

The other four methods produced results in the same direction as those of the IVW method. However, no significant causal effects were observed for increasing levels of PM2.5 and the other 17 types of cardiovascular diseases (Fig. 1, Table S2).

**Figure 4:**
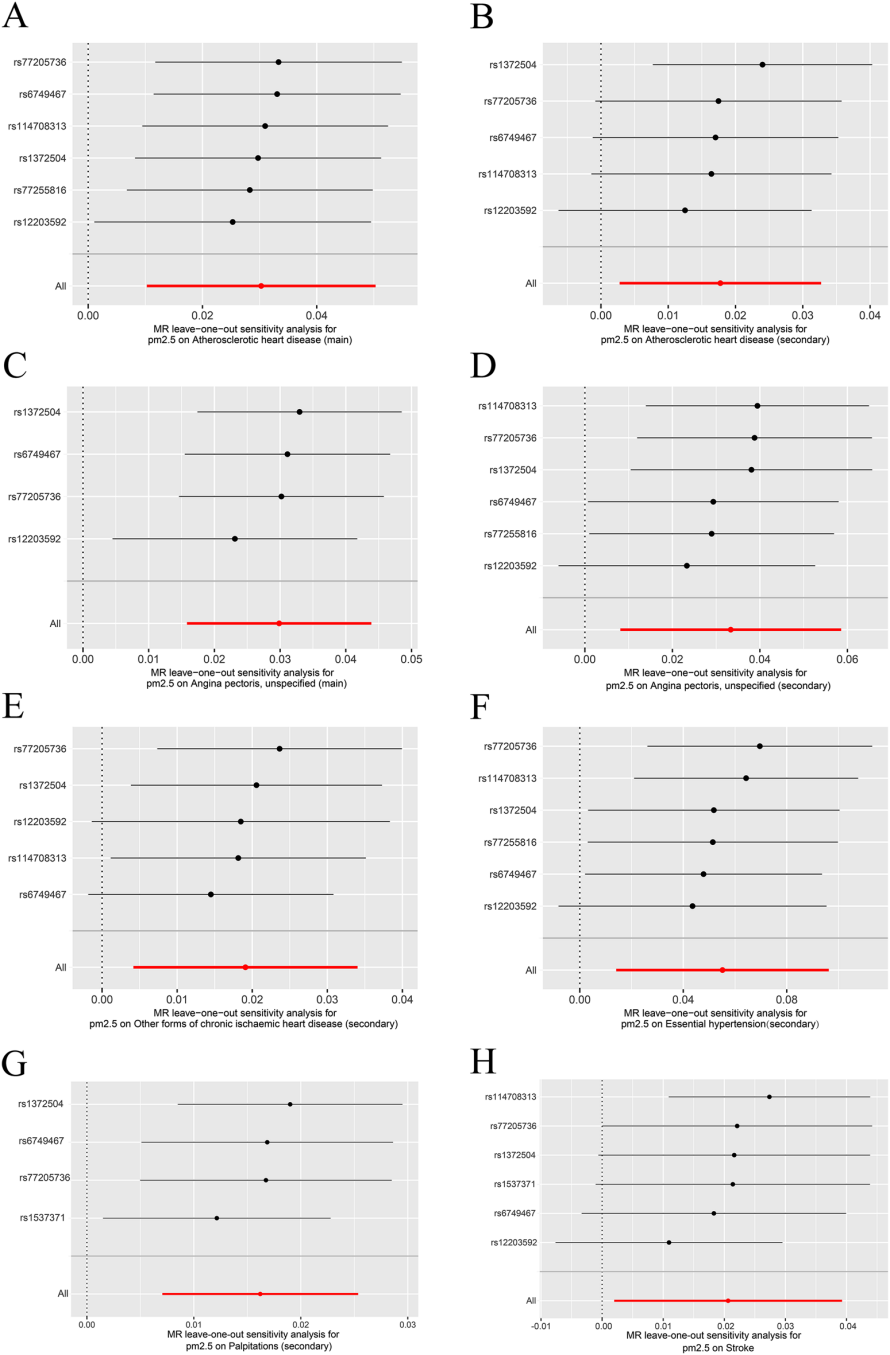
Leave-one-out sensitivity analysis displaying the Mendelian randomization analysis. (**A**) Atherosclerotic heart disease, main, (**B**) Atherosclerotic heart disease, secondary, (**C**) Angina pectoris unspecified, main**, **(**D**) Angina pectoris unspecified, secondary**, **(**E**) Other forms of chronic ischaemic heart disease, secondary**, **(**F**) Essential hypertension, secondary, (**G**) Palpitations, secondary**,** and (**H**) Stroke. MR, Mendelian randomization; SNP, single-nucleotide polymorphism.

To assess the reliability of our findings, we performed a series of sensitivity analyses, including Cochran's Q test, MR-Egger intercept test, and MR-PRESSO test. Heterogeneity emerged in the analysis of the association between PM2.5 and the risk of a diagnosis of Chronic ischaemic heart disease unspecified, main (Cochran's Q MR-Egger p = 0.0034) and Acute MI unspecified, main(Cochran's Q MR-Egger p = 0.0470) (Table S3) so the IVW random effects model was used. The rest of the analyses used the IVW fixed model. Furthermore, no substantial horizontal pleiotropy in the IVs was detected via MR-PRESSO, and no significant outlier was identified. Additionally, the p-values of all MR-Egger intercept results were above 0.05 (Table S3), indicating the absence of pleiotropy in these IVs. The leave-one-out analysis showed that a single instrumental variable was not driving the causal effect (Fig. 4). The F-values for all IVs were greater than 10, indicating that they were not weak instruments (Table S4). These provide additional support for the robustness and validity of our findings.

## Discussion

To the best of our current understanding, this study marks the initial foray into investigating the relationship between PM2.5 exposure and the incidence of cardiovascular diseases through MR analysis. Employing a two-sample MR approach, our study rigorously evaluated the causal impact of PM2.5 on the risk of 25 types of cardiovascular diseases. The outcomes underscore a notable correlation, demonstrating that heightened PM2.5 concentrations significantly elevate the risk of ASHD and Angina pectoris, both in primary and secondary diagnoses. Furthermore, there is an increased susceptibility to Other Forms of Chronic Ischaemic Heart Disease, Essential Hypertension, Palpitations as secondary diagnoses, and Stroke. The robustness of our conclusions was corroborated by F-values exceeding 10 for all IVs utilized in the MR analysis, further validated through meticulous sensitivity analyses.

Several studies, including those by Pope 3rd et al.^24^ and Yusuf et al.^25^, have consistently established a correlation between PM2.5 exposure and increased incidence and mortality rates in various cardiovascular conditions, such as ASHD, angina, chronic ischemic heart disease, hypertension, and stroke. Our study findings harmonize with these previous results, offering further insights. Specifically, our research indicates that heightened PM2.5 concentrations are linked to an elevated risk of primary and secondary diagnoses of ASHD and angina pectoris. This observation suggests that PM2.5 exposure does not exhibit bias towards increasing the risk of these two heart diseases. In essence, regardless of the baseline cardiovascular health of the population, exposure to PM2.5 heightens the risk of developing these diseases. Furthermore, our findings propose that these two heart diseases may be the primary cardiovascular conditions influenced by PM2.5 exposure^26,27^. On the contrary, other cardiovascular diseases, such as various forms of chronic ischemic heart disease, essential hypertension, and palpitations, were observed as secondary diagnoses associated with an increased risk due to PM2.5 exposure. This implies that PM2.5 might contribute more significantly to the occurrence of these diseases in specific populations. These populations may include individuals with characteristics like advanced age, diabetes, preexisting heart and lung diseases, and a history of smoking, factors that generally elevate the risk for cardiovascular diseases^28^. Previous studies have also emphasized that the elderly face a higher risk of adverse health effects related to short-term PM exposure^29^. Additionally, while chronic exposure to PM2.5 escalates the overall mortality rate of ischemic heart disease in the general population, it significantly amplifies the risk of death from arrhythmia, heart failure, and cardiac arrest specifically in smokers, but not in non-smokers^30^. These findings elucidate the complex relationship between PM2.5 exposure, diverse cardiovascular diseases, and specific risk factors, contributing valuable insights to the existing body of knowledge.

ASHD stands as a chronic cardiovascular condition characterized by the formation of atherosclerotic plaques along the inner walls of blood vessels^31^. The initial symptom of ASHD is angina pectoris, which arises when the coronary arteries, responsible for supplying blood to the heart, are affected by ASHD, causing an imbalance between the myocardial oxygen supply and demand^32^. ASHD can lead to severe complications, including unstable angina and MI, both of which can be life-threatening. Unstable plaques have the potential to rupture, triggering platelet and coagulation factor aggregation, and forming a thrombus that might cause arterial stenosis or complete obstruction. Consequently, this can lead to myocardial ischemia, MI, or unstable angina^33^. MI occurs when prolonged myocardial ischemia results in the necrosis of myocardial cells^34^. A study by Kaufman et al. established a connection between increased PM2.5 concentration and the progression of coronary artery calcification, indicating an accelerated atherosclerosis process^35^.

Our study findings revealed that increased PM2.5 concentration is associated with an elevated risk of ASHD and angina pectoris. However, no causal effect was observed with unstable angina and MI, suggesting that PM2.5 might primarily influence the early formation of atherosclerotic plaques in ASHD and not significantly impact plaque stability. It is also possible that while PM2.5 may contribute to insufficient blood supply to the heart, its detrimental effects have not reached the threshold for MI or unstable angina^36^. A multi-center cohort study conducted in Europe indicated that an increase in PM2.5 concentration is linked to a higher risk of all-cause mortality, but not specifically cardiovascular disease mortality^37^. However, other studies have reported a significant association between PM2.5 and MI^38–41^, possibly due to the progression of ASHD in conjunction with other air pollutants. A meta-analysis of 34 studies revealed that, except ozone, all air pollutants were considered potential factors contributing to MI^42^.

Recent research by Lakey et al.^43^ has suggested a potential association between exposure to air pollutants, including PM2.5, and an increased risk of atrial fibrillation (AF). Zheng et al.^44^ also reported a significant link between PM2.5 concentration levels and daily hospitalizations for arrhythmia. The underlying mechanisms that contribute to this association may involve PM2.5-induced cardiac autonomic dysfunction, alterations in heart rate variability, systemic inflammation, and oxidative stress^44–46^. However, our study did not find a significant association between elevated PM2.5 concentrations and specific types of arrhythmias, although a noteworthy relationship was observed with palpitations. This result could be attributed to the limited number of arrhythmia types included in our analysis. Additionally, combining the analysis of AF and atrial flutter may have diluted the possibility of obtaining a significant association. Furthermore, the baseline level of PM2.5 concentration might influence the analysis results. For instance, Stockfelt et al.^47^ evaluated the impact of exposure to a median concentration of 9 μg/m^3^ PM2.5 at different time points on the incidence of AF and found no significant association between chronic air pollution exposure and AF incidence. The analysis speculated that there may be a certain concentration threshold for PM2.5-induced AF^48^. Regional differences in the relative risk of air pollution-related arrhythmias further support this possibility, with Asia exhibiting a more pronounced relative risk compared to Europe and North America, likely due to higher average concentrations of PM10 and PM2.5 in Asia^48^. In our study, we did not observe significant associations with specific cardiac diseases, which could be attributed to the threshold set in our analysis. The mean PM2.5 concentration in our dataset was 9.9919 µg/m^3^, with a standard deviation of 1.057 µg/m^3^. The current limit values for PM2.5 set by the European Union and the United States Environmental Protection Agency are 25 µg/m^3^ and 12 µg/m^3^, respectively, which are higher than the threshold utilized in our study^9^.

Our study findings indicate a correlation between increased concentrations of PM2.5 and an elevated risk of hypertension. This aligns with a recent meta-analysis that demonstrated an association between PM2.5 exposure, both short-term and long-term, and hypertension. Short-term exposure was linked to an increase in systolic blood pressure, while both short and long-term exposure were associated with elevated diastolic blood pressure^49^. The potential mechanisms through which PM2.5 contributes to hypertension include systemic inflammation and oxidative stress, activation of inflammatory cytokines leading to impaired endothelial function, heightened sympathetic nervous system activity, disruption of vascular homeostasis, increased peripheral resistance, and arterial remodeling^50–52^. In turn, elevated blood pressure contributes to the development of atherosclerosis^53,54^.

Regarding stroke, a recent meta-analysis of a large cohort study involving 137,148 participants with a follow-up period of 17.2 years has revealed a significant association between PM2.5 exposure and the incidence of stroke^9^, which is consistent with our study findings. The increased risk appears to be more pronounced for ischemic stroke compared to hemorrhagic stroke^55^. The relatively lower sensitivity of hemorrhagic stroke to PM2.5 is akin to observations from studies on exposure to tobacco smoke, where the impact of tobacco smoke on ischemic stroke outweighs its effect on hemorrhagic stroke^56^. PM2.5-induced changes in sympathetic nervous system activity can lead to vasoconstriction, increased vascular resistance, and reduced cerebral blood flow^57^. Hypertension is more prevalent in patients with ischemic stroke, which contributes to the heightened sensitivity of this subtype to PM2.5. Moreover, compared to patients with hemorrhagic stroke, those with ischemic stroke are typically older and exhibit more severe atherosclerosis, which may further contribute to the observed heightened sensitivity^56^.

It is worth noting that this study did not employ multiple testing correction methods. This decision was made based on the following considerations: applying multiple testing corrections, such as the Bonferroni correction, would significantly limit the causal association between PM2.5 and cardiovascular diseases. This limitation stands in stark contrast to existing epidemiological and foundational research findings^2,10–15^. The disparity arises because while multiple testing corrections effectively reduce Type I errors (the probability of incorrectly rejecting a true effect), they might simultaneously increase Type II errors (the probability of failing to detect a true effect)^58,59^. Furthermore, this suggests that multiple testing corrections may not always be applicable, especially in exploratory studies^60–62^. Considering the exploratory nature of our research aimed at uncovering novel correlations and effects, the use of multiple testing correction methods was deemed inappropriate for the objectives of this study.

It is important to acknowledge several limitations in our study. Firstly, our findings are based on a comprehensive analysis of GWAS data from the UK Biobank, which introduces the possibility of confounding factors and unmeasured variables. Therefore, caution is warranted when interpreting the causality of our results. Secondly, the datasets utilized in our study had limitations in terms of population subdivisions, such as age and sex. This limited our ability to perform more detailed analyses and identify vulnerable populations that may be more susceptible to PM2.5-related cardiovascular diseases. Future research should consider employing datasets with finer population segmentation to allow for more precise conclusions through stratified analysis. Lastly, it is important to note that our study population consisted exclusively of individuals of European ancestry, potentially limiting the generalizability of our findings. Genomic variations across different populations can result in varying effects of the same exposure and genetic factors. Therefore, if we intend to extend the applicability of our findings to other populations, it is imperative to validate them within those specific populations.

## Conclusion

In our investigation, we revealed a causal association between heightened PM2.5 exposure and increased incidences of ASHD (primary or secondary, OR [95% CI] 1.0307 [1.0103–1.0516], p-value = 0.003 and OR [95% CI] 1.0179 [1.0028–1.0333], p-value = 0.0202) and Angina pectoris (primary or secondary, OR [95% CI] 1.0303 [1.0160–1.0449], p-value = 3.04e−05 and OR [95% CI] 1.0339 [1.0081–1.0603], p-value = 0.0096). Moreover, elevated PM2.5 exposure amplified the likelihood of diagnoses such as Other forms of chronic ischaemic heart disease (secondary, OR [95% CI] 1.0193 [1.0042–1.0346], p-value = 0.0121), Essential hypertension (secondary, OR [95% CI] 1.0567 [1.0142–1.1010], p-value = 0.0085), Palpitations (OR [95% CI] 1.0163 [1.0071–1.0257], p-value = 5e−04), and Stroke (OR [95% CI] 1.0208 [1.0020–1.0401], p-value = 0.0301). This highlights the pressing need for policymakers and healthcare professionals to prioritize interventions aimed at reducing PM2.5 concentrations, resulting in improved public health outcomes and decreased societal and economic costs associated with cardiovascular disease.

### Supplementary Information

Supplementary Legends.

Supplementary Figure S1.

Supplementary Tables.

## Data Availability

The datasets generated and/or analysed during the current study are available in the IEU open GWAS project (https://gwas.mrcieu.ac.uk/).
