# Author Correction: Two-sample mendelian randomization analysis investigates ambient fine particulate matter's impact on cardiovascular disease development

**DOI:** 10.1038/s41598-023-48867-y

**Published:** 2023-12-15

**Authors:** Xiao Liang, Lianjing Liang, Yuchao Fan

**Affiliations:** 1 Department of Anesthesiology, West China Hospital, Sichuan University, Chengdu, Sichuan Province China; 2 Department of Emergency Medicine, West China Hospital, Sichuan University, Chengdu, Sichuan Province China; 3 Department of Anesthesiology, Sichuan Clinical Research Center for Cancer, Sichuan Cancer Hospital and Institute, Sichuan Cancer Center, Affiliated Cancer Hospital of the University of Electronic Science and Technology of China, No. 55, Section 4, Renmin South Road, Chengdu, 610041 Sichuan Province China

Correction to: *Scientific Reports* 10.1038/s41598-023-46816-3, published online 17 November 2023

The original version of this Article contained an error in the name of Lianjing Liang, which was incorrectly given as Lianjin Liang.

The original Article has been corrected.

