# Supplementary material for: Two-sample mendelian randomization analysis investigates ambient fine particulate matter's impact on cardiovascular disease development
Source: Sci Rep. 2023 Nov 17;13:20129. doi: 10.1038/s41598-023-46816-3 (PMC10656567; doi:10.1038/s41598-023-46816-3)
Supplement: Supplementary file 1 — Supplementary Legends. [file 41598_2023_46816_MOESM1_ESM.docx]

Figure legend for the supplementary figure S1

**Figure S1.** **Mendelian randomization study design examining the causal effect of PM2.5 increasing on risk of cardiovascular diseases, requiring a genetic variant that meets three criteria:** 1. The genetic variant is associated with the exposure. 2. The genetic variant is not associated with any confounders of the exposure-outcome association. 3. The genetic variant does not affect outcome, except possibly through association with exposure. MR, Mendelian randomization.
