# Supplementary figures and images for: Two-sample mendelian randomization analysis investigates ambient fine particulate matter's impact on cardiovascular disease development
Source: Sci Rep. 2023 Nov 17;13:20129. doi: 10.1038/s41598-023-46816-3 (PMC10656567; doi:10.1038/s41598-023-46816-3)

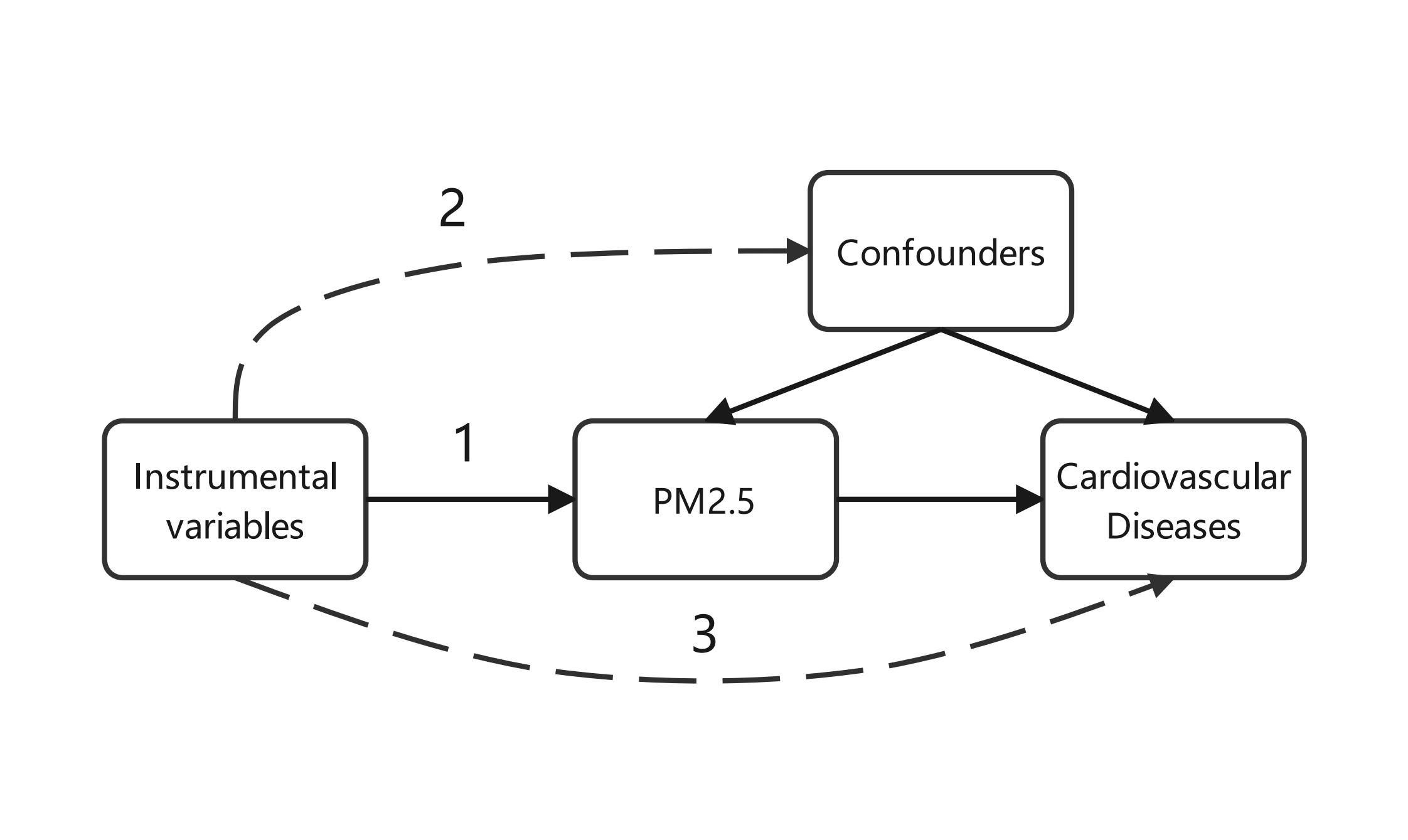

Supplement: Supplementary file 2 — Supplementary Figure S1. [file 41598_2023_46816_MOESM2_ESM.jpg]
